# Physics-informed, phenotype-based endotamponade selection in vitreoretinal surgery: current agents and emerging vitreous substitutes

**DOI:** 10.1186/s40942-026-00878-3

**Published:** 2026-06-16

**Authors:** Miguel A. Quiroz-Reyes, Erick A. Quiroz-Gonzalez, Miguel A. Quiroz Gonzalez, Virgilio Lima-Gomez

**Affiliations:** 1 Oftalmología Integral ABC,Retina Division (Medical and Surgical Nonprofit Organization), Affiliated With the Postgraduate Studies Division, National AutonomousUniversity of Mexico, Av. Paseo de las Palmas 735, Suite 303, Lomas de Chapultepec, Mexico City, 11000 Mexico; 2 Coordinating Commission of National Institutes of Health and High-Specialty Hospitals, Periférico Sur 4809, 6th Floor, Col. Arenal Tepepan, Mexico City, 14610 Mexico

**Keywords:** Endotamponade, Retinal detachment, Macular hole, Silicone oil, Heavy silicone oil, Perfluorocarbon liquid

## Abstract

**Background:**

Endotamponade selection is best understood as a physics-informed, phenotype-based decision rather than one driven mainly by tamponade duration or material class. This narrative review integrates density, interfacial tension, contact angle, viscosity, and drug–tamponade interactions with phenotype-specific clinical outcomes. The result is a practical framework linking tamponade behavior to retinal geography, disease complexity, and postoperative feasibility.

**Main body:**

Lighter-than-water agents (air, expansile gases, and light silicone oils [LSOs]) preferentially support the superior retina, whereas heavy silicone oils (HSOs) and perfluorocarbon liquids (PFCLs) provide stronger inferior support. In uncomplicated rhegmatogenous retinal detachment (RRD) and many idiopathic macular holes (MHs), air or sulfur hexafluoride (SF₆) usually achieves high anatomical success with less early intraocular pressure (IOP) elevation and cataract burden than longer-acting gas or silicone oil when brief postoperative positioning is feasible. LSO remains the principal long-term option for complex, tractional, and traumatic disease because it provides durable, relatively posture-independent support, but at the cost of emulsification, cataract, pressure-related, and corneal complications. HSO and staged short-term PFCL are best viewed as selective strategies for inferior-predominant giant retinal tear (GRT) or posture-limited phenotypes, where the mechanistic rationale is strong but comparative evidence remains largely observational. Hydrogel-based vitreous substitutes are promising but remain investigational.

**Conclusions:**

Current evidence supports a physics-informed, phenotype-based approach to endotamponade selection. Future research should prioritize phenotype-stratified comparative studies with standardized anatomical and emulsification endpoints and rigorous evaluation of emerging vitreous substitutes in well-defined clinical settings.

**Trial registration:**

Not applied.

**Supplementary Information:**

The online version contains supplementary material available at 10.1186/s40942-026-00878-3.

## Background

Endotamponade selection is most effective when treated as a physics-informed, phenotype-based decision. In practice, retinal geography, interface behavior, and postoperative feasibility shape outcomes more directly than tamponade duration or material class alone. Gases, perfluorocarbon liquids (PFCLs), and silicone oils (SOs) are central to modern vitrectomy for rhegmatogenous retinal detachment (RRD), macular hole (MH), and other complex vitreoretinal disorders. However, their value depends on how well their physicochemical behavior matches disease configuration and postoperative constraints [1–4].

Prior reviews usually discuss tamponades by agent class or broad diagnosis. Less often do they explicitly connect tamponade physics to clinically relevant retinal phenotypes [1, 3, 5–7]. This review addresses that gap by showing how density, interfacial tension (IFT), contact angle, viscosity, and drug–tamponade interactions translate into phenotype-specific performance across common vitreoretinal scenarios. Its aim is not to rank agents by material class, but to offer a practical framework for matching tamponade behavior to retinal phenotype [8–11].

Definitions of complex rhegmatogenous retinal detachment (RRD) and anatomical success vary across studies. In this study, complex RRD is defined as RRD associated with one or more of the following: proliferative vitreoretinopathy (PVR) grade C or higher, giant retinal tear (GRT), significant inferior pathology (multiple or large inferior retinal breaks), proliferative diabetic retinopathy (PDR)–related tractional retinal detachment (TRD), choroidal detachment, high myopia, aphakia, ocular trauma, or previous unsuccessful RRD surgery [1–5, 12–16]. For RRD, primary anatomical success is defined as complete retinal reattachment after a single operation with maintenance of the intended tamponade at final follow‑up. In contrast, final anatomical success is defined as complete retinal reattachment after any additional reoperations during follow‑up [1–5, 12]. For macular holes (MHs), anatomical success is defined as hole closure on optical coherence tomography (OCT) [5, 12, 17]. These criteria are not universally standardized and instead reflect operational definitions derived from prevailing usage in the contemporary literature [1, 2, 18].

Each tamponade entails trade‑offs. Prolonged SO tamponade is strongly associated with cataract formation, glaucoma, keratopathy, and emulsification; cataracts develop in up to 80–90% of phakic eyes, and keratopathy or secondary glaucoma in roughly 20–30% (moderate‑certainty observational evidence) [1–4, 19]. Heavy silicone oils (HSOs) were developed to improve inferior support, but most comparative studies show final anatomical outcomes similar to light silicone oils (LSOs) and do not establish routine superiority; several cohorts instead report greater early IOP elevation and anterior inflammation with HSOs (low‑certainty comparative evidence) [13, 18, 20, 21]. Recurrent retinal detachment (re-RD) after silicone oil removal (SOR) occurs in approximately 12–20% of eyes, particularly in aphakia, high myopia, prior failed RRD surgery, or trauma (moderate‑certainty observational evidence) [2, 4, 22]. Reports of silicone oil–related visual loss (SORVL) and microstructural changes indicate that tamponades exert biological effects beyond mechanical support [5, 6, 19, 23, 24].

A more useful framework focuses on the physicochemical properties that determine intraocular performance. Density and buoyancy determine whether the superior or inferior retina is preferentially supported. IFT, contact angle, and viscosity shape the meniscus and influence emulsification. Drug–tamponade interactions further modify pharmacokinetics and interface stability [8–10, 12, 13, 17, 25–33]. Together, these factors explain why agents with similar nominal indications differ in positioning demands, optical burden, complication patterns, and suitability for specific retinal phenotypes.

The limitations of current tamponades have driven the development of hydrogel-based vitreous substitutes, semifluorinated alkane (SFA)-containing oils, and drug-responsive systems designed to provide more posture-independent support and controllable drug delivery with fewer long-term complications [6, 7, 27, 34, 35]. Early preclinical data are encouraging, but robust clinical evidence remains sparse; it is still unclear in which settings these materials provide a meaningful advantage over established agents (low-certainty clinical evidence) [6, 34, 35].

### Scope, novelty, and contribution of this review

Mechanistic data are linked to clinical outcomes across major scenarios—superior and inferior RRD, MHs, PVR, GRTs, PDR‑related TRD, combined TRD–RRD, and trauma—to build an evidence‑graded decision pathway that matches agents to phenotypes rather than to material class. This review contributes in four main ways. First, it frames endotamponade selection around retinal phenotype rather than material class. Second, it integrates tamponade physics, clinical outcomes, and drug–tamponade interactions into a single, decision‑oriented framework. Third, it distinguishes recommendations supported by stronger clinical evidence from those that are mechanistically persuasive yet still clinically provisional. Finally, it situates emerging vitreous substitutes within current practice rather than in future promise alone.

## Methods

### Design and search

This narrative, decision-oriented review was based on a structured literature search; it was not conducted as a formal systematic review or meta-analysis. We searched MEDLINE/PubMed, Cochrane CENTRAL, and ClinicalTrials.gov for English language studies published from January 1, 2005, through September 19, 2025. A focused update on April 30, 2026, identified no new studies that changed the overall conclusions. At least two authors independently screened studies and abstracted data, and disagreements were resolved by consensus. We also hand-searched the reference lists of key reviews, meta-analyses, and landmark primary studies.

Foundational bench and modeling studies on tamponade physics were included without a start date restriction when they addressed clinically used agents or core physical principles. The search combined terms for tamponade agents, indications, and outcomes/physics domains; an example PubMed string is provided in Supplementary File 1.

### Eligibility and appraisal

We included randomized controlled trials (RCTs), nonrandomized comparative studies, cohort studies, case series with at least 10 patients, and existing systematic reviews and meta‑analyses. Mechanistic, bench, and modeling studies were eligible when they evaluated clinically relevant tamponades and reported quantitative physicochemical parameters or intraocular distribution.

We excluded single case reports and very small series unless they provided major safety or mechanistic insights; editorials without primary data; conference abstracts without full reports; clearly irrelevant animal studies; pediatric‑only series without broad adult relevance; obsolete agents unless they clarified core principles; and reports lacking usable outcome data or adequate tamponade details.

We did not apply a formal risk‑of‑bias tool but prioritized RCTs, systematic reviews, meta‑analyses, and larger comparative cohorts. Smaller retrospective and noncomparative series were interpreted with caution, especially when case mix, surgeon experience, follow‑up duration, or reporting completeness could introduce bias. When evidence conflicted, we gave greater weight to study design, phenotype comparability, consistency across studies, and relevance to current practice.

To improve transparency, we used an explicit, qualitative GRADE-inspired certainty framework that distinguishes clinical certainty (high, moderate, low) from mechanistic certainty for physicochemical data. High clinical certainty generally reflected consistent randomized or meta-analytic evidence in well-defined phenotypes. Moderate certainty reflected reasonably consistent comparative or observational evidence with important limitations. Low certainty reflected sparse, heterogeneous, or strongly selection-sensitive data. High mechanistic certainty was assigned when independent bench, modeling, and experimental studies converged on the same physical principle. Because most endotamponade data are observational, reported success and complication rates should be interpreted as approximate ranges that show relative patterns rather than precise benchmarks.

### Physicochemical foundations of intraocular tamponade agents

This section outlines the physical variables that determine how tamponades behave in the eye and why those behaviors matter clinically. The goal is to connect core physicochemical principles to retinal coverage, interface stability, emulsification, optical effects, and drug behavior.

#### Density and buoyancy

Gaseous tamponades and LSOs, with a density of approximately 0.97 g/cm^3^, are less dense than aqueous humor and therefore rise within the vitreous cavity. They exert their greatest tamponading effect on superior retinal regions and are particularly useful for treating superior retinal breaks and detachments [12, 13, 17, 32, 33].

In contrast, HSOs, with a density of 1.02–1.06 g/cm^3^ and PFCLs (1.7–1.9 g/cm^3^), are denser than aqueous solutions and sink to the inferior portion of the eye. Their higher specific gravity provides sustained mechanical support for inferior retinal breaks, extended retinectomies, and complex inferior PVR, enhancing retinal stabilization and promoting reattachment in these challenging cases [12, 13, 17, 32, 33].

Modeling and chamber studies show that even high‑volume LSO fills offer only limited inferior contact during typical eye movements. In contrast, HSOs and PFCLs maintain larger, more continuous inferior contact areas across gaze positions [13, 17].

**Clinical implications:** Lighter-than-water intraocular tamponade agents are well-suited to uncomplicated superior RRDs and many MHs when appropriate postoperative positioning is feasible. In these settings, primary anatomical success rates typically range from the high 80s to the mid-90s, supported by moderate certainty clinical evidence [5, 10, 12, 14, 17, 22, 36, 37].

By contrast, HSO and staged PFCL are used mainly for complex inferior RRDs, PVR with inferior extension, and GRTs with inferior components, particularly when strict facedown positioning is unlikely. Although the clinical evidence for these heavier agents is generally low to moderate in certainty, their use is supported by high mechanistic certainty in these challenging inferior pathologies [6, 13, 16–18, 21].

#### Interfacial tension and contact angle: interface stability and break coverage

IFT and contact angle are key determinants of how the tamponade–aqueous interface behaves along the retinal surface, and thus of interface stability and break coverage [8, 9, 17, 26, 29]. Lower IFT—for example, from blood, serum proteins, or intravitreal drugs—destabilizes the interface, promoting droplet formation and emulsification. High contact angles can also “pin” the meniscus, altering its configuration and shifting which retinal regions receive effective tamponade. These effects are especially relevant in eyes with multiple or eccentric retinal breaks, where small interface changes can materially affect tamponade performance.

**Clinical implications**: Emulsification risk is driven more by interface contamination (blood, protein, drugs) than by label viscosity alone. Eyes with significant hemorrhage or repeated intravitreal therapy under oil warrant closer monitoring and often earlier SOR.

#### Viscosity and emulsification

Shear viscosity mainly affects the intraoperative handling of tamponade agents, whereas extensional viscosity is more important for their behavior at the tamponade–aqueous interface [8–12]. Higher viscosity can slow emulsification, but does not eliminate it. In contrast, amphiphilic molecules and other contaminants strongly promote and stabilize droplet formation, even when bulk viscosity is largely unchanged [9, 10, 29].

**Clinical implications:** Choosing a higher‑viscosity oil may slow emulsification, but it does not eliminate it. Meticulous removal of blood and debris and achieving a clean, near‑complete fill are at least as important as viscosity grade in reducing emulsification‑related complications.

#### Drug partitioning and interface stability

In vitro, bevacizumab remains primarily in the aqueous phase in SO‑ and HSO‑filled models, whereas hydrogels and native vitreous act as drug reservoirs, smoothing peaks and prolonging exposure [7, 27, 28, 34, 35]. Bevacizumab also lowers silicone–water IFT, potentially destabilizing the interface [9, 26–29]. Triamcinolone accumulates at the silicone–water interface rather than in the bulk oil [7, 27, 28].

**Clinical implications**: In oil‑filled eyes, anti‑VEGF agents and corticosteroids do not behave as they do in native vitreous. Onset and duration may differ, and repeated injections may need closer follow‑up and individualized intervals.

#### Optical and practical considerations

SO, with a refractive index of about 1.405, induces a myopic shift that can alter intraocular lens (IOL) power calculations and degrade visual quality [5, 12, 19]. PFCLs, with a refractive index of 1.27–1.33, offer excellent intraoperative visualization but are unsuitable for long‑term use due to retinal toxicity and intraocular inflammation [7, 25, 33, 38–40]. Emulsified water‑in‑oil droplets scatter light and can cause substantial visual disturbance; when they migrate into the anterior chamber, they are linked to corneal endothelial loss and keratopathy [14, 19, 24, 31].

**Clinical implications**: Anticipate refractive shifts and potential visual disturbance in oil‑filled eyes; adjust refraction, counseling, and timing of oil removal accordingly. Monitor the cornea closely and consider earlier SOR if anterior droplets or prolonged endothelial contact develop.

## Traditional tamponades and core clinical data

### Gases

For uncomplicated RRD with predominantly superior breaks, air or short‑acting gas (usually SF₆) achieves primary reattachment rates of 85–95%, comparable to longer‑acting gas or SO, but with fewer early IOP spikes and less cataract progression (moderate‑ to high‑certainty clinical evidence) [5, 12, 22, 41, 42].

For many idiopathic MHs, air or SF₆ also achieves high closure rates, without consistent evidence that longer‑acting gas is superior in routine phenotypes [5, 12, 17]. Longer‑acting gases (C₃F₈) are mainly reserved for large, chronic, highly myopic, or posture‑limited MHs.

C₂F₆ is an intermediate‑duration expansile gas whose behavior lies between SF₆ and C₃F₈; it is used less commonly in contemporary practice, with fewer dedicated comparative data than SF₆ or C₃F₈ [5, 12, 17, 41–44]. In this review, C₂F₆ is therefore not discussed in detail and is instead considered within the same physics‑based framework as a medium‑duration, lighter‑than‑water gas tamponade that provides more sustained superior support than SF₆ at the cost of greater postoperative visual burden and gas‑related precautions [5, 12, 17, 41, 42].

### Light silicone oils

LSOs (commonly 1,000–5,000 cSt) remain the principal long‑term option for complex RRD, PVR, PDR‑related TRD, and trauma because they provide durable, relatively posture‑independent support [2–5, 12]. Across observational cohorts, final anatomical success often exceeds 90%.

Morbidity includes emulsification (typically after 5–24 months), cataract progression in most phakic eyes, and residual droplets after SOR that are associated with later IOP abnormalities and glaucoma treatment (moderate‑certainty clinical evidence) [2–4, 14, 15, 23]. Aphakia, high myopia, prior failed surgery, and trauma increase re‑RD risk after SOR; prophylactic 360° laser or scleral buckling may reduce risk in selected high‑risk eyes (low‑certainty clinical evidence) [2, 4, 22].

### Heavy silicone oils

HSOs (e.g., Densiron 68, Oxane HD) were developed to improve inferior retinal support in complex RRD, particularly with inferior PVR, inferior GRT components, or extensive inferior retinectomies [13, 16, 18, 20, 21, 32, 37]. Their higher density improves inferior coverage and can reduce dependence on strict face‑down positioning (high mechanistic certainty) [13, 17].

Observational cohorts and multicenter series generally show primary success at least as high as LSO in very complex, inferior-predominant RRD, but final reattachment rates converge after reoperations [13, 16, 18, 20, 21, 37]. Several series report earlier IOP elevation and anterior inflammation with HSO (low to moderate-certainty clinical evidence) [13, 16, 18, 20, 21]. HSOs are therefore best used as selective tools for clearly inferior, predominant, or posture-limited phenotypes rather than as routine tamponades.

### Perfluorocarbon liquids

PFCLs are indispensable intraoperative tools because their high density stabilizes the posterior pole, flattens the retina, and counteracts posterior traction, facilitating GRT, extended retinectomies, and complex tractional detachments [7, 25, 33, 38–40, 45]. They displace subretinal fluid anteriorly and help prevent retinal slippage.

Long-term PFCL use is precluded by inflammatory and toxic risk. Selected retrospective series support very short-term staged use (typically 7–10 days) in GRT repair and severe inferior pathology, with final anatomical success around 90–93% (low-certainty clinical evidence) [38–40, 45]. Planned exchange is mandatory.

A plot of representative endotamponade densities versus water at approximately 25 °C is shown in Figure S1 of Supplementary File 2.

### Emulsification, IOP, and tissue safety of intraocular silicone oil endotamponades

Emulsification is driven mainly by interfacial instability and contamination (blood, proteins, amphiphilic drugs), not by oil “degradation” [8–11, 15, 24, 26, 29]. Clinically, a greater droplet burden is associated with IOP elevation, uveitis, keratopathy, and later glaucoma therapy; residual droplets persist in most eyes after SOR [2–4, 10, 14, 15, 19, 23, 24, 31]. Long-term SO tamponade increases the risk of keratopathy, uveitis, retinal changes, optic neuropathy, and cataract formation [2–4, 10, 14, 15, 19, 24, 31]. The key silicone-related risk-reduction strategies are summarized in Table 1.

**Table 1:** Practical strategies to reduce siliconerelated morbidity

**Domain**	**Practical strategy**	**Specific aim**
Pre‑/intraoperative	Minimize intraocular blood/protein/debris before oil fill	Reduce emulsification‑promoting contamination.
	Use high‑purity silicone oil; avoid mixing oils	Stabilize interface behavior.
	Aim for a high, stable fill (90–100%)	Maximize retinal coverage and interface stability.
	Inject/exchange oil gently (low turbulence)	Limit initial droplet formation.
	Use 360° laser retinopexy or buckle only in very high-risk eyes	Lower re‑RD risk in selected phenotypes.
Postoperative monitoring	Monitor IOP closely, especially early and if emulsification appears	Enable timely treatment and SOR.
	Regular slit‑lamp ± AS‑OCT/ultrasound for droplets	Detect emulsification and anterior migration early.
	Frequent corneal checks in aphakia/compromised anterior segments	Catch keratopathy/endothelial damage early.
Anterior segment protection	Maintain inferior PI when indicated	Prevent pupillary block and anterior oil migration.
	Avoid prolonged supine positioning	Limit endothelial contact.
	Remove significant anterior chamber oil promptly	Reduce endothelial toxicity and IOP spikes.
SOR timing	Tailor oil dwell time to retinal status, PVR, axial length, IOP, cornea	Balance re‑RD risk vs cumulative morbidity.
	Bring SOR forward with emulsification, uncontrolled IOP, or keratopathy	Limit long‑term silicone‑related damage.
	Plan combined/staged cataract surgery in likely progressors	Improve visualization; avoid extra procedures.
SOR technique	Meticulous anterior and posterior washout	Minimize residual droplets and later IOP issues.
	Clear angle/anterior chamber of residual oil	Reduce angle compromise and inflammation.
	Combine SOR with glaucoma surgery when IOP is refractory	Improve long‑term pressure control.
Drug considerations	Use caution with agents that lower interfacial tension (e.g., anti‑VEGF)	Avoid further destabilizing the interface.
	Adjust intravitreal dosing under oil vs native vitreous	Reflect altered pharmacokinetics.

Note: AS-OCT, anterior segment optical coherence tomography; IOP, intraocular pressure; VEGF, vascular endothelial growth factor; PI, peripheral iridectomy; PVR, proliferative vitreoretinopathy; re-RD, recurrent retinal detachment; SOR, silicone oil removal

### Indication-specific tamponade selection, surgical optimization, and clinical application

**Fig. 1:**
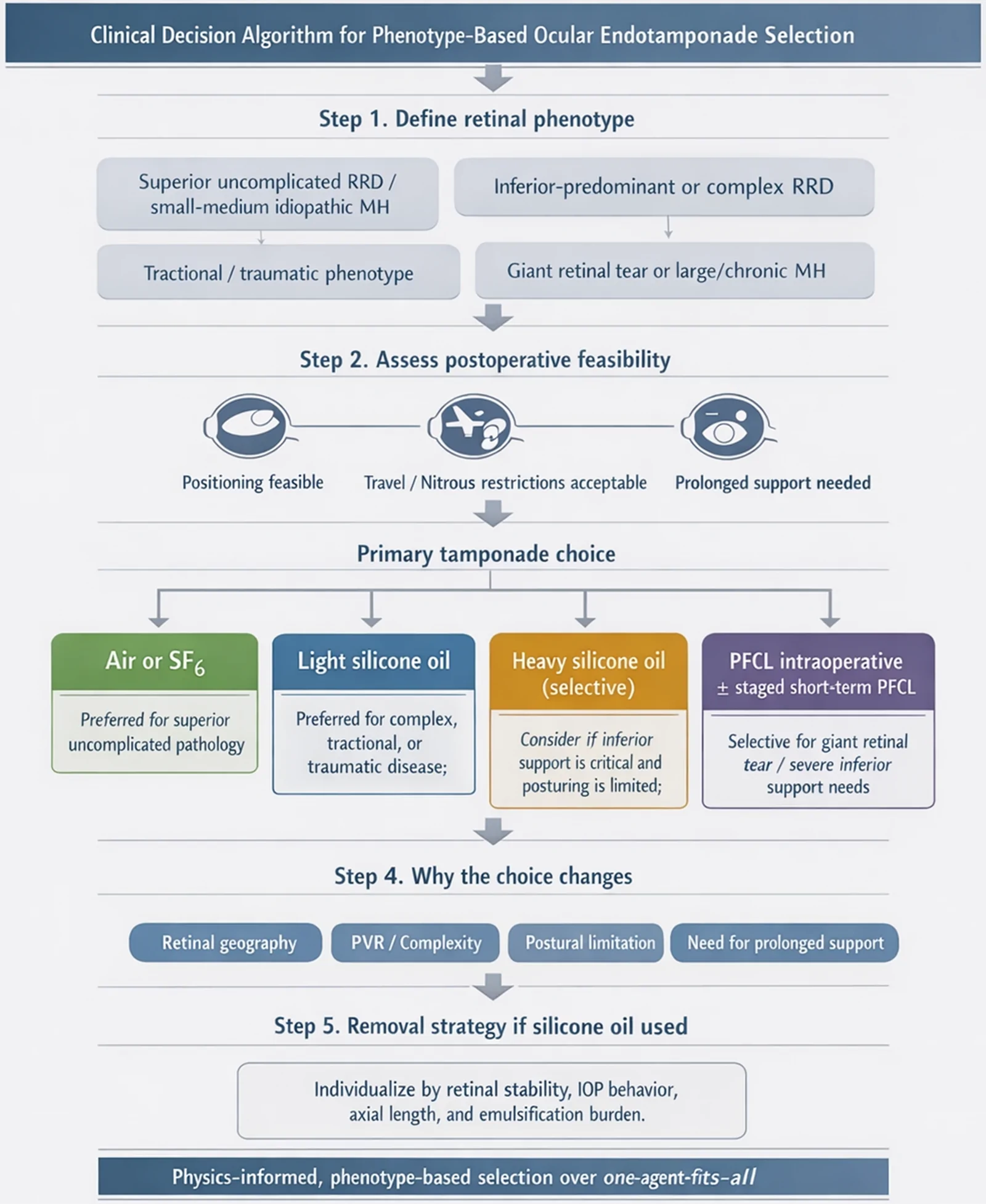
Phenotype-based algorithm for endotamponade selection. Clinical decision pathway linking retinal geography, disease complexity, and postoperative feasibility to preferred endotamponade strategies. The algorithm emphasizes a physics-informed, phenotype-based approach rather than a material hierarchy. Branches supported mainly by observational data (low- to moderate-certainty clinical evidence) should be interpreted as evidence-informed guidance rather than prescriptive recommendations

This section translates the mechanistic and clinical evidence into practical selection by matching tamponade behavior to retinal geography, disease complexity, and postoperative feasibility. Table 2 summarizes the main phenotype-based choices, trade-offs, and evidence certainty, and Fig. 1 outlines the broader decision pathway for common vitreoretinal scenarios. Because the underlying data are largely observational and heterogeneous, the algorithm should be read as evidence-informed guidance rather than a validated prescriptive tool.

**Table 2:** Concise clinical decision summary for endotamponade selection

Phenotype/scenario	Preferred tamponade strategy	Rationale	Main trade-offs/cautions	Evidence certainty
Uncomplicated superior RRD	Air or short acting gas (usually SF₆)	Strong superior support with high primary reattachment rates and faster visual recovery.	Requires postoperative positioning; longer-acting gas may add visual burden and IOP risk without clear routine benefit.	Moderate- to high-certainty clinical evidence
Inferior-predominant or more complex RRD	LSO as default; HSO selectively	LSO provides durable, relatively posture-independent support; HSO may improve inferior coverage when posturing is limited.	LSO carries emulsification and removal-related morbidity; HSO has earlier IOP and inflammatory complications.	Moderate-certainty clinical evidence for LSO; low- to moderate-certainty clinical evidence for selective HSO use
Giant retinal tear or severe inferior pathology requiring staged support	PFCL intraoperatively; short-term staged PFCL in selected cases	Provides strong inferior stabilization and flap control during or immediately after surgery.	Not suitable for long-term tamponade because of inflammatory and toxic risk; requires planned exchange.	Low-certainty clinical evidence; high mechanistic certainty
Idiopathic macular hole, small to medium size	Air or short acting gas	High closure rates with lower postoperative burden and faster recovery than longer-acting gas in routine phenotypes.	Less sustained support in larger, chronic, or highly myopic holes.	Moderate- to high-certainty clinical evidence
Large, chronic, highly myopic, or posture-limited macular hole	Longer-acting gas (often C₃F₈); silicone oil selectively	Provides more sustained support when closure risk is higher or positioning is difficult.	Greater positioning demands, slower recovery, and more limited comparative evidence.	Low- to moderate-certainty clinical evidence
PDR-related TRD, combined TRD–RRD, or severe trauma	LSO as default; HSO selectively for marked inferior disease	Offers durable support and better globe stability in ischemic or structurally unstable eyes.	High complication burden with prolonged oil exposure; HSO advantages remain phenotype-specific rather than generalizable.	Moderate-certainty clinical evidence for LSO; low- to moderate-certainty clinical evidence for selective HSO use

The subsections that follow explain how this framework applies to the most common retinal phenotypes encountered in practice.

#### Primary rhegmatogenous retinal detachment

**Uncomplicated superior RRD:** Air or short‑acting gas (usually SF₆) is generally preferred. It preserves high primary reattachment rates while reducing early IOP elevation, cataract burden, and postoperative visual disability versus longer‑acting gas or SO [5, 12, 22, 41–44].

**Selected inferior break RRD:** Can sometimes be repaired with air or short‑acting gas when vitreous base shaving, retinopexy, and postoperative positioning are meticulous, but evidence is lower in certainty and likely influenced by selection [36, 41, 42, 45].

**More extensive inferior or complex pathology:** Silicone‑based support becomes more defensible. LSO is the default long‑term option; HSO is reserved for markedly inferior‑predominant cases or when maximizing inferior coverage and reducing strict posturing is prioritized despite earlier IOP and inflammatory complications [1–5, 12, 13, 16, 18, 20, 21, 37].

#### Inferior and complex retinal detachment

For inferior and complex detachments, LSO remains the default long‑term strategy because it provides durable, relatively posture‑independent support across a wide range of high‑risk phenotypes (moderate‑certainty clinical evidence) [2–5, 12, 14–16].

HSOs can be considered when inferior disease predominates, and postoperative positioning is limited, because their higher density improves inferior coverage and reduces reliance on strict posturing (high mechanistic certainty; low to moderate clinical evidence certainty) [13, 16–18, 20, 21, 37]. In the large multicenter matched cohort by Tzoumas et al. (2024), HSO modestly improved primary anatomical success versus LSO in very complex primary RRD, but final success converged after reoperations [21]. Several series also report higher rates of early IOP elevation and anterior inflammation with HSO [13, 16, 18, 20, 21]. HSOs, therefore, remain selective rather than routine.

#### Giant retinal tears

PFCL‑assisted vitrectomy remains standard for stabilizing GRT flaps and flattening the retina [7, 17, 25, 33]. In selected cases, short‑term postoperative PFCL (typically 7–10 days before exchange with gas or SO) may reduce flap slippage, with final reattachment rates around 90–93% in retrospective series (low‑certainty clinical evidence) [38–40, 45]. Longer dwell times increase the risk of inflammation and toxicity.

#### Macular hole

**Small‑ to medium‑sized idiopathic MHs (≤400–500 μm):** Vitrectomy with ILM peeling and air or short‑acting gas usually achieves closure rates of 90–98%, with faster visual recovery and less postoperative burden than longer‑acting gas [5, 12, 17].

**Large, chronic, highly myopic, or posture-limited MHs:** Closure rates decline as diameter, chronicity, and axial length increase. Many surgeons favor longer-acting expansile gas (often C₃F₈) or, selectively, SO to provide more sustained support. Evidence is largely observational (low to moderate-certainty clinical evidence) [5, 12, 17].

#### PDR-related TRD, combined detachments, and trauma

In PDR-related TRD, combined TRD–RRD, and severe trauma, SO is generally preferred because it provides durable, relatively posture-independent support and helps preserve the globe configuration in ischemic or structurally unstable eyes [3, 5, 12, 13, 16–18, 20, 21]. Across observational series, final reattachment rates in these high-risk phenotypes typically range from 85 to 95% under SO, with clinically significant IOP elevation in about 15–30% of eyes and cataract progression in roughly 70–90% of phakic eyes during followup [1–5, 12, 14–16, 19].

### Managing complications within an integrated, physics-informed approach

The main tamponade-related complications are emulsification, IOP elevation, glaucoma, keratopathy, cataract formation, and inflammatory responses [1–4, 14, 15, 19, 31].

**IOP elevation and glaucoma:** Early pressure spikes are especially common in HSO‑filled eyes [2–4, 10, 13–16, 18, 20, 21, 24, 37]. Management ranges from topical/systemic therapy to glaucoma surgery. Persistent IOP elevation, particularly with emulsification or angle compromise, should prompt consideration of earlier SOR and coordinated glaucoma procedures [2–4, 14, 15, 22–24].

**Keratopathy:** Most likely when oil or droplets contact the corneal endothelium, especially in aphakia or compromised anterior segments [3, 14, 19, 24, 31]. Prevention and management include maintaining a patent inferior peripheral iridotomy (PI) when appropriate, avoiding prolonged supine positioning, and promptly evacuating anterior chamber oil. Advanced cases may require endothelial keratoplasty once the posterior segment is stable.

**Cataract formation:** Cataract progression is expected in many phakic eyes exposed to prolonged SO and should be addressed in preoperative counseling and surgical planning [1, 2, 14, 19]. Combined or staged cataract surgery should be tailored to retinal stability, visualization needs, and overall risk [2–5, 12, 17].

**Inflammatory complications and PVR:** These are exacerbated by emulsified droplets, hemorrhage, and intraocular debris, especially in trauma, extensive retinotomy, and repeat surgery [3, 5, 15, 19, 31]. Key strategies include meticulous intraoperative cleaning, hemorrhage control, structured postoperative surveillance, and selective use of adjuncts such as 360° laser, encircling bands, or prolonged tamponade in very highrisk eyes, given the observational and heterogeneous evidence [2–4, 14–16, 23].

## Discussion

This discussion integrates the physical principles and clinical evidence into a single decision framework. It highlights where the evidence is strongest, where it remains mainly mechanistic or observational, and which questions should guide future comparative studies.

### Integrated interpretation of the evidence

Taken together, the evidence supports phenotype-matched selection rather than a search for a universally superior tamponade. Once the choice is aligned with retinal geography, disease complexity, and postoperative feasibility, the key differences are retinal coverage, posture dependence, emulsification risk, optical burden, and removal strategy [1–5, 12–14, 16, 17, 37]. In practice, gases remain first-line for uncomplicated superior pathology; LSO remains the principal long-term option for complex or tractional disease; and HSO or staged short-term PFCL are reserved for clearly defined inferior-predominant, GRT, or posture-limited phenotypes, where comparative evidence remains limited.

High clinical certainty is concentrated in randomized and meta‑analytic comparisons of short‑acting gases versus longer‑acting gases or SO in uncomplicated RRD and idiopathic MH [5, 12, 17, 22, 41, 42]. By contrast, most evidence for LSO in complex disease, HSO in inferior‑predominant phenotypes, and staged PFCL strategies remains observational, so complication patterns and postoperative logistics are more reliably distinguished than headline final attachment rates.

**Clinical implications:** Choose tamponade based on retinal phenotype, prioritizing the best match to retinal geography, disease complexity, and realistic postoperative feasibility.

### Physics-based determinants and emulsification as drivers of silicone oil morbidity

A small set of physical variables explains most clinically relevant differences in tamponade performance. Density determines whether the superior or inferior retina is preferentially supported. IFT and contact angle govern interface stability, three-phase line behavior, and break coverage. Viscosity can modulate interface failure, but it does not prevent it in the presence of contamination or inflammation [5, 6, 8–13, 17, 25–29]. Across bench experiments, model eye systems, and computational fluid dynamics studies, these relationships are highly consistent. Together, they provide high mechanistic certainty that buoyancy and interfacial phenomena dominate intraocular behavior [8–11, 17, 26, 29, 30].

Within this framework, HSOs illustrate the gap between mechanistic plausibility and clinical certainty. Their higher density (1.02–1.06 g/cm^3^) provides a clear buoyancy-driven rationale for improved inferior retinal support and reduced reliance on strict postoperative positioning in inferior-predominant phenotypes (high mechanistic certainty) [13, 17]. Large observational cohorts, including the multicenter matched study by Tzoumas et al. (2024), suggest a modest advantage in primary anatomical success over standard LSO in very complex primary RRD. However, final reattachment rates generally converge once reoperations are included, and several series report more frequent early IOP elevation and anterior inflammation (low to moderate-certainty clinical evidence) [13, 16, 18, 20, 21, 37]. HSOs are therefore best regarded as selective adjuncts for well-defined inferior or posture-limited phenotypes rather than as inherently superior long-term tamponades.

Across silicone‑based strategies more broadly, emulsification and interfacial instability emerge as the main drivers of morbidity. Experimental work shows that proteins, blood, inflammatory debris, and amphiphilic drugs markedly lower silicone–aqueous IFT and promote droplet formation at the moving three‑phase contact line, particularly under eye‑like shear [8–11, 17, 26, 29, 30]. Clinical series consistently associate a greater burden of emulsified droplets with elevated IOP, anterior uveitis, keratopathy, and subsequent glaucoma therapy, and residual droplets persist in most eyes after SOR [2–4, 10, 14, 15, 19, 23, 24, 31]. These convergent mechanistic and clinical data (moderate‑certainty clinical evidence; high mechanistic certainty) support a management focus on interface control rather than viscosity grade or nominal dwell time alone.

**Clinical implications:** Use HSOs selectively for inferior-predominant or posture-limited phenotypes. Prioritize near-complete fills, clean silicone–aqueous interfaces, individualized SOR timing, and meticulous droplet clearance at removal to reduce IOP elevation, uveitis, keratopathy, and glaucoma.

### Technical and postoperative strategies to reduce complications

Technical and postoperative management should be individualized rather than tied to fixed removal intervals. The main determinants are retinal stability, PVR severity, axial length, prior failure, IOP behavior, and early corneal or inflammatory change. Adjunctive measures such as prophylactic 360° laser retinopexy, encircling bands, or prolonged tamponade may help in carefully selected very high-risk eyes, but the supporting evidence is conflicting and largely observational.

Prophylactic 360° laser retinopexy and encircling buckles before SOR remain evidence-informed expert practices rather than guideline-level standards, because available data are retrospective, heterogeneous, and strongly influenced by selection bias [2–5, 14–16, 22, 23]. These measures are best reserved for selected high-risk phenotypes rather than applied routinely.

**Clinical implications:** Individualize SOR timing and postoperative strategy based on retinal stability, PVR severity, axial length, prior failure, IOP behavior, and early corneal or inflammatory changes. Reserve adjunctive measures such as 360° laser retinopexy, encircling bands, and prolonged tamponade for clearly defined high-risk phenotypes.

### Pharmacologic implications under existing tamponades

Intravitreal anti-VEGF agents and corticosteroids do not behave the same way under silicone-based tamponades as they do in native vitreous. Experimental studies consistently show altered partitioning, accumulation at the silicone–aqueous interface, and reduced interface stability in oil-filled eyes [7, 9, 26–28]. However, the clinical magnitude of these effects remains uncertain because human data are limited and mostly observational. Repeated intravitreal treatment in oil-filled eyes should therefore be individualized rather than assumed to follow native vitreous dosing patterns.

These interactions are most relevant when repeated therapy is delivered under silicone tamponade. Bevacizumab remains largely in the aqueous phase and may accumulate at the silicone–aqueous interface, whereas triamcinolone may concentrate at the interface rather than distribute uniformly; closer follow-up and individualized retreatment intervals may therefore be needed [7, 9, 27, 28]. Direct comparative clinical data remain limited.

**Clinical implications:** Do not assume that intravitreal regimens validated in native vitreous transfer directly to oil-filled eyes. Altered drug partitioning, diffusion, and clearance may require reassessment of agent choice, dosing intervals, and retreatment thresholds.

### Investigational replacement platforms

In response to the pharmacologic and interfacial limitations of existing silicone tamponades, investigational platforms—including thermosetting and stimuli-responsive hydrogels—aim to provide more stable bulk support and more predictable intravitreal drug behavior than buoyancy- and interface-dependent agents [7, 34, 35]. Their proposed advantages include broader retinal support, greater posture independence, and more controllable pharmacologic behavior. However, they remain experimental because long-term data on safety, optical performance, reversibility, removal, and cost-effectiveness are limited. A platform-level overview is provided in Table S1 of Supplementary File 3.

**Clinical implications:** Hydrogel vitreous substitutes remain experimental; no platform is currently approved for routine long-term intraocular tamponade in standard clinical practice, so tamponade selection should continue to rely on physics-informed, phenotype-based reasoning applied to established agents, acknowledging the limits of heterogeneous, largely nonrandomized evidence.

A central strength of this review is that it links physicochemical principles directly to phenotype-specific clinical choice. It also distinguishes the strong mechanistic evidence for buoyancy and interface behavior from the weaker comparative clinical evidence available for many complex phenotypes [1–5, 8, 10–12, 14–18, 20, 21, 30, 38, 39]. A key limitation is that published studies use heterogeneous definitions of complexity, inferior involvement, and anatomical success, and often do not report outcomes in the phenotype strata most relevant to tamponade selection. For that reason, the framework should be read as a structured aid to clinical reasoning rather than as a validated scoring system or a substitute for phenotype-specific comparative trials.

## Conclusion

Endotamponade selection is best framed as a physics-informed, phenotype-based decision that integrates retinal geography, disease complexity, and postoperative feasibility. In this model, gases remain the first-line option for uncomplicated superior pathology and many idiopathic MHs; LSO remains the principal long-term option for complex, tractional, and traumatic disease; and HSO or staged short-term PFCL are selective tools for clearly defined inferior-predominant, GRT, or posture-limited phenotypes. Future work should prioritize phenotype-stratified comparative studies with standardized definitions of complexity and anatomical success, clinically meaningful emulsification endpoints, and rigorous longterm evaluation of emerging vitreous substitutes.

## Electronic supplementary material

Below is the link to the electronic supplementary material.

Supplementary Material 1

Supplementary Material 2

Supplementary Material 3

## Data Availability

No datasets were generated or analysed during the current study.

## Abbreviations

^AS‑OCT^: Anterior segment optical coherence tomography
^anti‑VEGF^: Anti‑vascular endothelial growth factor
^BEAVRS^: British and Eire Association of Vitreoretinal Surgeons
^BDDE^: 1,4‑butanediol diglycidyl ether
^Bond number^: Dimensionless fluid dynamics parameter
^C₂F₆^: Hexafluoroethane
^C₃F₈^: Perfluoropropane
^CI^: Confidence interval
^cSt^: Centistokes
^EVRS^: European Vitreoretinal Society
^GRT^: Giant retinal tear
^HA^: Hyaluronic acid
^HMW^: High molecular weight
^HSO^: Heavy silicone oil
^IFT^: Interfacial tension
^ILM^: Internal limiting membrane
^IOP^: Intraocular pressure
^LSO^: Light silicone oil
^MH^: Macular hole
^OCT^: Optical coherence tomography
^OR^: Odds ratio
^PDMS^: Polydimethylsiloxane
^PEG^: Polyethylene glycol
^PFCL^: Perfluorocarbon liquid
^PFO^: Perfluoro‑N‑octane
^PFD^: Perfluorodecalin
^PDR^: Proliferative diabetic retinopathy
^PVR^: Proliferative vitreoretinopathy
^RCT^: Randomized controlled trial
^re‑RD^: Recurrent retinal detachment
^RRD^: Rhegmatogenous retinal detachment
^SF₆^: Sulfur hexafluoride
^SFA^: Semifluorinated alkane

## Declarations

### AI assisted technologies

During manuscript preparation, the authors used ChatGPT (OpenAI) for language editing and to improve clarity. The authors reviewed and edited all AI‑generated content and take full responsibility for the final manuscript.
