# Supplementary material for: Physics-informed, phenotype-based endotamponade selection in vitreoretinal surgery: current agents and emerging vitreous substitutes
Source: Int J Retina Vitreous. 2026 Jun 16;12:111. doi: 10.1186/s40942-026-00878-3 (PMC13504771; doi:10.1186/s40942-026-00878-3)
Supplement: Supplementary file 1 [file 40942_2026_878_MOESM1_ESM.docx]

**Supplementary file 1.** Search strategy

**Search strategy.** We combined MeSH and free‑text terms for tamponade agents, indications, and outcomes. Agents included gases (air, SF₆, C₂F₆, C₃F₈), SOs (polydimethylsiloxane [PDMS], Densiron, Oxane HD), and PFCLs (perfluoro‑n‑octane [PFO], perfluorodecalin [PFD]). Indications included RRD, PVR, GRT, MH, TRD/PDR, and trauma. Outcome/physics terms included emulsification, interfacial tension, viscosity, contact angle, IOP, keratopathy, uveitis, toxicity, cataract, positioning, fill, removal, success, and recurrence.

An example PubMed search was: (“silicone oil” OR PDMS OR “heavy silicone oil” OR Densiron OR “Oxane HD” OR PFCL OR “perfluoro‑n‑octane” OR PFO OR perfluorodecalin OR PFD OR air OR SF₆ OR C₂F₆ OR C₃F₈) AND (“retinal detachment” OR RRD OR PVR OR “giant retinal tear” OR GRT OR “macular hole” OR TRD OR PDR OR trauma) AND (“emulsification” OR “interfacial tension” OR viscosity OR “contact angle” OR meniscus OR position OR posture OR fill OR removal OR IOP OR glaucoma OR keratopathy OR uveitis OR toxicity OR cataract OR outcome OR success OR recurrence).
