# Supplementary material for: Physics-informed, phenotype-based endotamponade selection in vitreoretinal surgery: current agents and emerging vitreous substitutes
Source: Int J Retina Vitreous. 2026 Jun 16;12:111. doi: 10.1186/s40942-026-00878-3 (PMC13504771; doi:10.1186/s40942-026-00878-3)
Supplement: Supplementary file 2 [file 40942_2026_878_MOESM2_ESM.docx]

**Supplementary file 2. Figure S1.** Endotamponade density vs water

**Figure 1.** Bar plot of representative endotamponade densities at approximately 25 °C. The dashed line indicates water (1.0 g/mL). Gases (air, SF₆, C₃F₈) were well below 1.0 g/mL (appearing near zero on this scale), indicating superior coverage. Light silicone oils (PDMS 1000/5000 cSt) cluster at approximately 0.97 g/mL, indicating superior coverage. Heavy silicone oils (Oxane HD =1.02; Densiron 68 = 1.06 g/mL) provide inferior coverage. Perfluorocarbon liquids (PFO =1.76; PFD =1.94 g/mL) provide strongly inferior support for intraoperative use. The values are typical at room temperature; clinical behavior depends on temperature, fill, posture, and ocular geometry. Abbreviations: HSO, heavy silicone oil; LSO, light silicone oil; PFCL, perfluorocarbon liquid; PDMS, polydimethylsiloxane.
