# Supplementary material for: Physics-informed, phenotype-based endotamponade selection in vitreoretinal surgery: current agents and emerging vitreous substitutes
Source: Int J Retina Vitreous. 2026 Jun 16;12:111. doi: 10.1186/s40942-026-00878-3 (PMC13504771; doi:10.1186/s40942-026-00878-3)
Supplement: Supplementary file 3 [file 40942_2026_878_MOESM3_ESM.docx]

## **Supplementary File 3. Table S1**

**Table S1. Reference summary of established and emerging endotamponade platforms**

| **Agent/platform** | **Distinctive properties** | **Important cautions** | **Development status / typical role** |
| --- | --- | --- | --- |
| Air / SF₆ | Low density, strong superior buoyancy, fast visual recovery, short intraocular duration. | Requires positioning; inadequate for many inferior or prolonged-support phenotypes; gas-specific travel and pressure constraints apply. | Established first-line superior tamponades for routine clinical use. |
| Longer‑acting gas (C₃F₈) | Prolonged superior support and slower absorption than SF₆. | Greater visual burden, stricter positioning demands, and more prolonged gas precautions. | Established option when sustained gas support is intentionally prioritized. |
| Light silicone oil (LSO) | Long-term, relatively posture-independent support; available in multiple viscosity grades; accommodates unstable or ischemic eyes. | Emulsification, cataract, IOP elevation, keratopathy, residual droplets, and a planned removal procedure remain major limitations. | Established long-term platform and current default for many complex phenotypes. |
| Heavy silicone oil (HSO) | Heavier‑than‑water blends designed to improve inferior retinal coverage and reduce dependence on strict face‑down positioning. | More early IOP elevation and inflammation; comparative outcome advantage over LSO is inconsistent. | Established but selective adjunct platform for clearly inferior‑predominant or posture‑limited phenotypes. |
| PFCL | Very high density with strong inferior stabilization and flap control; excellent intraoperative manipulation properties. | Not suitable for prolonged postoperative tamponade because of inflammatory and toxic risk; requires planned exchange if staged. | Established intraoperative tool; staged short-term postoperative use remains selective. |
| Hydrogel-based vitreous substitutes | Biomimetic bulk gels with tunable viscoelasticity, potential posture independence, and drug-delivery flexibility. | Long-term safety, reversibility, optical performance, and cost-effectiveness remain insufficiently defined. | Investigational and not yet routine clinical substitutes. |

Note: LSO, light silicone oil; HSO, heavy silicone oil; PFCL, perfluorocarbon liquid; RRD, rhegmatogenous retinal detachment; IOP, intraocular pressure; SF₆, sulfur hexafluoride; C₃F₈, perfluoropropane.
